# Supplementary material for: Effects of xylo-oligosaccharide and flavomycin on the immune function of broiler chickens
Source: PeerJ. 2018 Mar 5;6:e4435. doi: 10.7717/peerj.4435 (PMC5842763; doi:10.7717/peerj.4435)
Supplement: Figure S1 — The actual P-values of Short-chain fatty acid concentrations. [file peerj-06-4435-s001.docx]

Short-chain fatty acid concentrations

Acetate at 21 days

|  | mean | SD | p value | letter |
| --- | --- | --- | --- | --- |
| CTL | 49.88 | 2.19 | CTL-FLA, P<0.001 | b |
| FLA | 42.05 | 2.50 | CTL-XOS, P=0.003 | c |
| XOS | 55.20 | 2.04 | FLA-XOS, P<0.001 | a |

Acetate at 42 days

|  | mean | SD | p value | letter |
| --- | --- | --- | --- | --- |
| CTL | 59.12 | 2.88 | CTL-FLA, P<0.001 | b |
| FLA | 42.54 | 3.80 | CTL-XOS, P<0.001 | c |
| XOS | 74.75 | 5.95 | FLA-XOS, P<0.001 | a |

Propionate at 21 days

|  | mean | SD | p value | letter |
| --- | --- | --- | --- | --- |
| CTL | 8.15 | 0.57 | CTL-FLA, P=0.001 | b |
| FLA | 10.19 | 0.55 | CTL-XOS, P=0.081 | a |
| XOS | 9.02 | 0.97 | FLA-XOS, P=0.025 | b |

Propionate at 42 days

|  | mean | SD | p value | letter |
| --- | --- | --- | --- | --- |
| CTL | 7.31 | 1.00 | CTL-FLA, P<0.001 | b |
| FLA | 12.87 | 2.73 | CTL-XOS, P=0.146 | a |
| XOS | 9.03 | 0.81 | FLA-XOS, P=0.005 | b |

Butyrate at 21 days

|  | mean | SD | p value | letter |
| --- | --- | --- | --- | --- |
| CTL | 6.97 | 0.63 | CTL-FLA, P=0.958 | b |
| FLA | 6.99 | 0.34 | CTL-XOS, P<0.001 | b |
| XOS | 9.81 | 0.73 | FLA-XOS, P<0.001 | a |

Butyrate at 42 days

|  | mean | SD | p value | letter |
| --- | --- | --- | --- | --- |
| CTL | 8.07 | 0.02 | CTL-FLA, P=0.799 | b |
| FLA | 8.17 | 0.02 | CTL-XOS, P<0.001 | b |
| XOS | 11.31 | 0.01 | FLA-XOS, P<0.001 | a |
