# Supplementary material for: Effects of xylo-oligosaccharide and flavomycin on the immune function of broiler chickens
Source: PeerJ. 2018 Mar 5;6:e4435. doi: 10.7717/peerj.4435 (PMC5842763; doi:10.7717/peerj.4435)
Supplement: Figure S2 — The actual P-values of the relative mRNA expression of immune genes. [file peerj-06-4435-s002.docx]

The relative mRNA expression of immune genes

*IFNγ* at 21 days

|  | mean | SD | p value | letter |
| --- | --- | --- | --- | --- |
| CTL | 1.00 | 0.07 | CTL-FLA, P<0.001 | a |
| FLA | 0.53 | 0.04 | CTL-XOS, P<0.001 | c |
| XOS | 0.65 | 0.01 | FLA-XOS, P=0.002 | b |

*IFNγ* at 42 days

|  | mean | SD | p value | letter |
| --- | --- | --- | --- | --- |
| CTL | 1.00 | 0.02 | CTL-FLA, P<0.001 | a |
| FLA | 0.75 | 0.04 | CTL-XOS, P<0.001 | b |
| XOS | 0.75 | 0.02 | FLA-XOS, P=0.997 | b |

*LITAF* at 21 days

|  | mean | SD | p value | letter |
| --- | --- | --- | --- | --- |
| CTL | 1.00 | 0.02 | CTL-FLA, P<0.001 | a |
| FLA | 0.72 | 0.06 | CTL-XOS, P<0.001 | b |
| XOS | 0.70 | 0.03 | FLA-XOS, P=0.363 | b |

*LITAF* at 42 days

|  | mean | SD | p value | letter |
| --- | --- | --- | --- | --- |
| CTL | 1.00 | 0.03 | CTL-FLA, P<0.001 | a |
| FLA | 0.76 | 0.02 | CTL-XOS, P<0.001 | c |
| XOS | 0.81 | 0.04 | FLA-XOS, P=0.013 | b |

*TLR5* at 21 days

|  | mean | SD | p value | letter |
| --- | --- | --- | --- | --- |
| CTL | 1.00 | 0.04 | CTL-FLA, P<0.001 | a |
| FLA | 0.60 | 0.03 | CTL-XOS, P<0.001 | c |
| XOS | 0.70 | 0.03 | FLA-XOS, P=0.001 | b |

*TLR5* at 42 days

|  | mean | SD | p value | letter |
| --- | --- | --- | --- | --- |
| CTL | 1.00 | 0.04 | CTL-FLA, P<0.001 | a |
| FLA | 0.77 | 0.03 | CTL-XOS, P<0.001 | b |
| XOS | 0.78 | 0.01 | FLA-XOS, P=0.404 | b |
