# Supplementary material for: Effects of xylo-oligosaccharide and flavomycin on the immune function of broiler chickens
Source: PeerJ. 2018 Mar 5;6:e4435. doi: 10.7717/peerj.4435 (PMC5842763; doi:10.7717/peerj.4435)
Supplement: Table S1 — The actual P-values of performance of broilers. [file peerj-06-4435-s003.docx]

Performance of broilers

ADFI for 1-21 days

|  | mean | SD | p value | letter |
| --- | --- | --- | --- | --- |
| CTL | 50.31 | 1.23 | CTL-FLA, P=0.292 | a |
| FLA | 51.31 | 1.66 | CTL-XOS, P=0.530 | a |
| XOS | 50.89 | 1.35 | FLA-XOS, P=0.656 | a |

ADG for 1-21 days

|  | mean | SD | p value | letter |
| --- | --- | --- | --- | --- |
| CTL | 32.95 | 0.79 | CTL-FLA, P=0.177 | a |
| FLA | 33.81 | 0.95 | CTL-XOS, P=0.427 | a |
| XOS | 33.44 | 1.07 | FLA-XOS, P=0.551 | a |

FCR for 1-21 days

|  | mean | SD | p value | letter |
| --- | --- | --- | --- | --- |
| CTL | 1.53 | 0.01 | CTL-FLA, P=0.326 | a |
| FLA | 1.52 | 0.01 | CTL-XOS, P=0.614 | a |
| XOS | 1.52 | 0.02 | FLA-XOS, P=0.622 | a |

ADFI for 1-42 days

|  | mean | SD | p value | letter |
| --- | --- | --- | --- | --- |
| CTL | 96.46 | 1.26 | CTL-FLA, P=0.089 | b |
| FLA | 98.36 | 1.59 | CTL-XOS, P=0.001 | b |
| XOS | 101.06 | 1.95 | FLA-XOS, P=0.022 | a |

ADG for 1-42 days

|  | mean | SD | p value | letter |
| --- | --- | --- | --- | --- |
| CTL | 50.29 | 1.04 | CTL-FLA, P=0.047 | b |
| FLA | 51.83 | 1.32 | CTL-XOS, P=0.001 | a |
| XOS | 53.25 | 0.88 | FLA-XOS, P=0.063 | a |

FCR for 1-42 days

|  | mean | SD | p value | letter |
| --- | --- | --- | --- | --- |
| CTL | 1.92 | 0.02 | CTL-FLA, P=0.080 | a |
| FLA | 1.90 | 0.02 | CTL-XOS, P=0.074 | a |
| XOS | 1.90 | 0.01 | FLA-XOS, P=0.963 | a |
