# Supplementary material for: Effects of xylo-oligosaccharide and flavomycin on the immune function of broiler chickens
Source: PeerJ. 2018 Mar 5;6:e4435. doi: 10.7717/peerj.4435 (PMC5842763; doi:10.7717/peerj.4435)
Supplement: Table S2 — The actual P-values of immune-related factors in the plasma. [file peerj-06-4435-s004.docx]

Immune-related factors in the plasma

IgG at 21 days

|  | mean | SD | p value | letter |
| --- | --- | --- | --- | --- |
| CTL | 2.03 | 0.16 | CTL-FLA, P=0.156 | b |
| FLA | 2.19 | 0.13 | CTL-XOS, P=0.002 | b |
| XOS | 2.45 | 0.21 | FLA-XOS, P=0.039 | a |

IgG for 42 days

|  | mean | SD | p value | letter |
| --- | --- | --- | --- | --- |
| CTL | 1.52 | 0.11 | CTL-FLA, P=0.035 | a |
| FLA | 1.31 | 0.11 | CTL-XOS, P=0.452 | b |
| XOS | 1.59 | 0.18 | FLA-XOS, P=0.008 | a |

IL-2 at 21 days

|  | mean | SD | p value | letter |
| --- | --- | --- | --- | --- |
| CTL | 170.65 | 9.32 | CTL-FLA, P=0.030 | a |
| FLA | 147.84 | 16.40 | CTL-XOS, P=0.914 | b |
| XOS | 171.67 | 16.97 | FLA-XOS, P=0.024 | a |

IL-2 at 42 days

|  | mean | SD | p value | letter |
| --- | --- | --- | --- | --- |
| CTL | 215.38 | 21.01 | CTL-FLA, P=0.002 | a |
| FLA | 158.27 | 24.01 | CTL-XOS, P=0.430 | b |
| XOS | 227.29 | 24.07 | FLA-XOS, P<0.001 | a |
